# Redox Status, Dose and Antioxidant Intake in Healthcare Workers Occupationally Exposed to Ionizing Radiation

**DOI:** 10.3390/antiox9090778

**Published:** 2020-08-21

**Authors:** Natividad Sebastià, Lorena Olivares-González, Alegría Montoro, Joan-Francesc Barquinero, Antonio José Canyada-Martinez, David Hervás, Pilar Gras, Juan Ignacio Villaescusa, Luis Martí-Bonmatí, Bianca Tabita Muresan, José Miguel Soriano, Juan Manuel Campayo, Joaquin Andani, Oscar Alonso, Regina Rodrigo

**Affiliations:** 1 Service of Radiological Protection, Clinical Area of Medical Image, Avda. Fernando Abril Martorell, 106, Hospital U. P. La Fe, 46026 Valencia, Spain; natividad.sebastia@uv.es (N.S.); almonpas@hotmail.com (A.M.); gras_pil@gva.es (P.G.); villaescusa_ign@gva.es (J.I.V.); bianca.muresan90@gmail.com (B.T.M.); juancampayo@gmail.com (J.M.C.); osalna@gmail.com (O.A.); 2 Biomedical Imaging Research Group GIBI230, Avda. Fernando Abril Martorell, 106, Health Research Institute Hospital La Fe (IISLaFe), 46026 Valencia, Spain; marti_lui@gva.es; 3 Pathophysiology and Therapies for Visual Disorders, Eduardo Primo Yúfera, 3, Research Center Príncipe Felipe (CIPF), 46012 Valencia, Spain; lolivares@cipf.es; 4 Center for Biomedical Network Research on Rare Diseases (CIBERER), Monforte de Lemos, 3-5. Pabellón 11, 28029 Madrid, Spain; 5 Biological Anthropology Unit Animal Biology, Plant Biology and Ecology Department, Universitat Autònoma de Barcelona (UAB), 08193 Bellaterra, Spain; francesc.barquinero@uab.cat; 6 Biostatistics Unit, Avda. Fernando Abril Martorell, 106, Health Research Institute Hospital La Fe (IISLaFe), 46026 Valencia, Spain; data_science@iislafe.es (A.J.C.-M.); bioestadistica@iislafe.es (D.H.); 7 Service of Radiology, Clinical Area of Medical Image, Avda. Fernando Abril Martorell, 106, Hospital U. P. La Fe, 46026 Valencia, Spain; 8 Food & Health Lab, Institute of Materials Science, Parc Científic, Catedrático Agustín Escardino, Paterna (Valencia), University of Valencia, 46980 Valencia, Spain; jose.soriano@uv.es; 9 Joint Research Unit on Endocrinology, Nutrition and Clinical Dietetics, University of Valencia, Avda. Fernando Abril Martorell, 106, Health Research Institute Hospital La Fe (IISLaFe), 46026 Valencia, Spain; 10 Service of Occupational Risk Prevention, Avda. Fernando Abril Martorell, 106, Hospital U. P. La Fe, 46026 Valencia, Spain; andani_joa@gva.es; 11 Joint Research Unit of Rare Diseases, CIPF-Health Research Institute La Fe, Eduardo Primo Yúfera, 3, 46012 Valencia, Spain

**Keywords:** antioxidant-oxidant status, ionizing radiation, occupational exposure, oxygen radical absorbance capacity (ORAC), personal dose equivalent

## Abstract

The purpose of this study was to evaluate the relationship between blood redox status, dose and antioxidant dietary intake of different hospital staff groups exposed to low doses of ionizing radiation (LDIR) (Interventional Radiology and Cardiology, Radiation Oncology, and Nuclear Medicine) and non-exposed. Personal dose equivalent (from last year and cumulative), plasma antioxidant markers (total antioxidant capacity, extracellular superoxide dismutase activity, and glutathione/oxidized glutathione ratio), oxidative stress markers (nitrites and nitrates, and lipid peroxidation) and dietary intake (antioxidant capacity using ORAC values) were collected and analyzed from 28 non-exposed healthcare workers and 42 healthcare workers exposed to LDIR. Hospital staff exposed to LDIR presented a redox imbalance in blood that seems to correlate with dose. Workers from the Nuclear Medicine Unit were the most affected group with the lowest value of plasma antioxidant response and the highest value of plasma thiobarbituric acid reactive substances, TBARS (indicator of lipid peroxidation) of all four groups. Cumulative personal dose equivalent positively correlated with nitrites and negatively correlated with total antioxidant capacity in blood. The diet of healthcare workers from Nuclear Medicine Unit had higher ORAC values than the diet of non-exposed. Therefore, occupational exposure to LDIR, especially for the Nuclear Medicine Unit, seems to produce an imbalanced redox status in blood that would correlate with cumulative personal dose equivalent.

## 1. Introduction

The evaluation of the health effects due to low doses of ionizing radiation (LDIR) in healthcare workers is a challenge. Ionizing radiation interacts with living cells, generating a wide range of highly reactive oxygen (ROS) and nitrogen (RNS) species which eventually damage macromolecules, such as DNA, proteins or lipids [1,2]. Affected cells counteract free radicals formation by antioxidant defense mechanisms allowing self-repairing, operating either normally or dysfunctionally, or dying [3,4]. Excessive and uncontrolled generation of free radicals leads to oxidative stress that contributes to the pathogenesis of several diseases, including neurodegenerative disorders, cancer, cardiovascular abnormalities, diabetes mellitus and liver damage. The free radical-mediated effect of ionizing radiation is suspected to have caused a majority of radiation-induced damage [5], and can trigger cell apoptosis or even genetic instability in living organisms [6]. Furthermore, inflammatory responses originated by ionizing radiation might further increase the risk of cancer development [7,8,9]. Therefore, the United Nations Scientific Committee on the Effects of Atomic Radiation (UNSCEAR) recommends to focusing on the evaluation of cancer and non-cancer diseases induced by exposure to LDIR [6,10]. Healthcare workers involved in diagnostic and/or treatment procedures with ionizing radiation represent a large collective exposed to long-term low or low radiation doses [11]. This collective includes physicians, medical physicists, nurses, and radiology technologists. There are some reports indicating that employees occupationally exposed to relatively low radiation doses at interventional radiology procedures suffer from a dis-regulated oxidative stress status [11,12], higher DNA breakage frequencies [13,14] and even an increase in cancer incidence, mainly leukemia [7].

Since exposure to very LDIR can lead to a broad range of consequences, it is worth exploring its health effects. The aims of this study are to assess whether healthcare workers occupationally exposed to low levels of ionizing radiation and to different conditions of the workplace present an altered redox status in blood compared to non-exposed workers and whether personal dose equivalent or antioxidant dietary intakes affect this blood redox status.

## 2. Materials and Methods

### 2.1. Selection and Description of Participants

In the present study, we enrolled 42 healthcare workers exposed to LDIR belonging to three hospital services of the University and Polytechnic La Fe Hospital (Valencia, Spain) and 28 healthcare workers non-exposed to ionizing radiation from the same hospital. Main demographic features of all are shown in Table 1.

The healthcare workers exposed to LDIR enrolled in this study, were classified into three groups with different levels of “occupational exposure to ionizing radiation” (Royal Decree 783/2001 according to the characteristics of their workplace, the groups were: Group 1: workers from the Interventional Radiology and Interventional Cardiology Service; Group 2: workers from the Radiation Oncology Service; Group 3: workers from Nuclear Medicine Service.

Personal whole-body and hand skin dose equivalent from last 12 months before to blood collection (Hp(10)_12_ and Hp(0.07)_12_ and cumulative personal dose equivalent (Hp(10)) were obtained from the personal dosimeters of these workers exposed to LDIR [15] (Table 1 and Table S1). The cumulative Hp(10) is computed by summing annual doses for the period during which the worker has been exposed to radiation. The mean ± standard error of the mean (SEM) of personal and hand skin dose equivalent for each group and individual data are presented in Table 1 and Table S1, respectively. Detection limit of the physical dosimetry is 0.1 mSv. Therefore a dose below this limit will be considered 0 mSv.

All participants were in good health and were not presently taking medications. All of them underwent a clinical survey that included questions related to lifestyles and medical records (age, medications, smoking, drinking habits, cholesterol and blood pressure) (Table 1).

Before peripheral blood collection, written informed consent was also obtained to participate in the study. The procedure complied with the Declaration of Helsinki and was approved by the Hospital Ethics Committee (Medicaments Research Ethics Committee-CEIm of the Health Research Institute Hospital La Fe (IIS La Fe)) (Reference 2012/0390).

### 2.2. Dietary Assessment and Dietary Antioxidant Capacity Estimation

Antioxidant dietary intake was assessed using a 24 h recall questionnaire at three different days (three questionnaires each participant) [16]. This questionnaire provides detailed information about all foods, beverages, dietary supplements, tobacco and alcohol consumption during 24 h and at three different days. It also allows identifying the existence of fruits and vegetables in the diet, the presence or absence of unhealthy food and signs of an antioxidant deficiency diet. Total antioxidant capacity of diet was calculated using the Oxygen Radical Absorbance Capacity (ORAC) scores of previously analyzed foods [17]. The ORAC value for each food was utilized to develop individual indices for dietary antioxidant capacity. ORAC values were reported in μmol of Trolox equivalents per gram (μmol TroloxE/g). Dietary antioxidant capacity describes the ability of food antioxidants to scavenge free radicals [18].

### 2.3. Blood Collection

Blood samples (10 mL) were collected in heparinized tubes and processed to obtain plasma by centrifugation at 800× *g* for 10 min. Plasma was stored in several aliquots at −80 °C until biochemical determinations as soon as possible (2–3 months). Freeze-thaw cycles were avoided.

### 2.4. Determination of Antioxidant and Oxidant Markers

Plasma antioxidant-oxidant (redox) status was evaluated by measuring total antioxidant capacity (TAC), lipid peroxidation (TBARS), extracellular SOD (EC-SOD) activity, nitrites and nitrates (NOX) levels, glutathione (GSH) and oxidized glutathione (GSSG) as previously described [19,20]. To avoid technical variations samples from non-exposed and exposed workers were always included for each determination. Specifically, TAC was measured by the ABTS ((2,2’-azino-bis(3-ethylbenzothiazoline-6-sulfonic acid)) method with a commercial kit (Cayman Chemical, Ann Arbor, MI, USA). Lipid peroxidation was evaluated by measuring the formation of thiobarbituric acid reactive substances (TBARS, including malondialdehyde (MDA) which are formed as a byproduct of lipid peroxidation (Cayman Chemical, Ann Arbor, MI, USA). Determination of EC-SOD activity was based on the dismutation of superoxide oxygen and hydrogen peroxide with a commercial kit (Cayman Chemical, Ann Arbor, MI, USA) Nitrites (stable end-product of NO) and nitrate (NOX) levels were measured by nitrate reductase and Griess reaction [21]. GSH and GSSG were measured by the liquid chromatography tandem mass spectrometry (LC-MS/MS) system [20].

### 2.5. Statistics

Statistical analyses were performed using R software (version 3.4.2) (foundation for statistical computing, Vienna, Austria). Normal distribution of data was analyzed by Shapiro–Wilk and Kolmogorov–Smirnov tests. Comparisons between non-exposed and exposed workers were performed using the Mann–Whitney U test (nonparametric analysis). Comparisons between non-exposed and different groups of exposed workers were performed using Kruskal–Wallis and Dunn’s multiple comparisons test or ANOVA-one way and post hoc Tukey depending on data distribution. Spearman’s rank correlation coefficient was used for non-parametric data. Ordinal regression models, which are a multivariable generalization of the Wilcoxon–Mann–Whitney test, were used to assess associations between the different groups and oxidative stress status, controlling for age, gender, ORAC, presence of high cholesterol and smoking habit by including them in the models as potential confounding factors. A fuzzy clustering algorithm was used to globally group the different individuals according to their oxidative stress status [22,23]. The probability of the workers to belong to a group with high (**1**, high TBARS/NOX and low TAC/EC-SOD values) or low (**0**, low TBARS/NOX and high TAC/EC-SOD values) oxidative stress was modeled. These values of 0 or 1 were regarded as their global oxidative stress level. Then, beta regression was used to assess differences in the fuzzy clustering results among the different groups. or beta regression, smoking habit, gender, age, ORAC and cumulative Hp(10) were included as confounding factors. *p* values < 0.05 were considered statistically significant.

## 3. Results

### 3.1. High Values of Personal Dose Equivalent and Dietary Antioxidant Capacity among Workers from Nuclear Medicine

Table 1 showed main characteristics of the participants. We analyzed whether values of personal and hand skin equivalent dose were different between healthcare workers exposed to LDIR belonging to different services. Kruskal–Wallis and Dunn’s multiple comparisons test revealed that healthcare workers from Nuclear Medicine Service (Group 3) showed higher values of Hp(10)_12_ than those from the Interventional Radiology and Interventional Cardiology Service (Group 1, *p* < 0.05) or from the Radiation Oncology Service (Group 2, *p* < 0.01) (Table 1).

We also analyzed whether ORAC values were different between healthcare workers non-exposed and exposed to LDIR. Values observed for workers of the Nuclear Medicine Service (Group 3) were significantly higher than those values from non-exposed (*p* < 0.05, ANOVA-one way and post hoc Tukey). For Groups 1 and 2 although ORAC values tended to be higher than in non-exposed workers, this difference was not statistically significant (*p* = 0.43 and *p* = 0.42, respectively, ANOVA-one way and post hoc Tukey) (Table 1).

### 3.2. Putative Correlation between Impaired Redox Status and Personal Dose Equivalent

**Figure 1:**
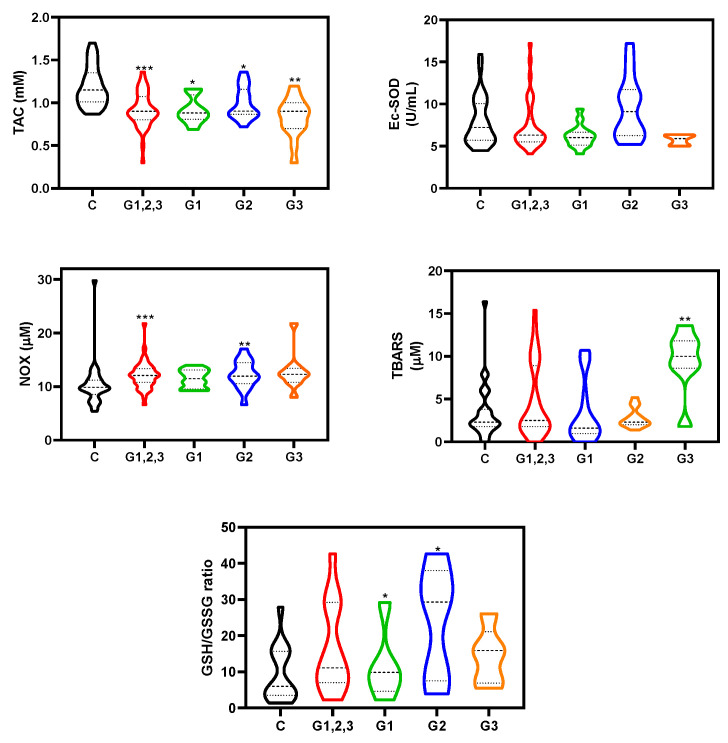
Plasma antioxidant-oxidant markers from healthcare workers occupationally exposed to low doses of ionizing radiation. Total antioxidant capacity (TAC), extracellular superoxide dismutase (EC-SOD) activity, thiobarbituric acid reactive substances (TBARS), nitrites (NOX) content and glutathione/oxidized glutathione ratio (GSH/GSSG) were determined from plasma as described in Material and Methods. C: Workers non-exposed to ionizing radiation; G1: Interventional Radiology and Cardiology; G2: Radiation Oncology; G3: Nuclear Medicine. Statistical analysis was carried out as described in Material and Methods. Ordinal regression models considering several variables simultaneously were used (* *p* < 0.05; ** *p* < 0.01; *** *p* < 0.001).

**Figure 2:**
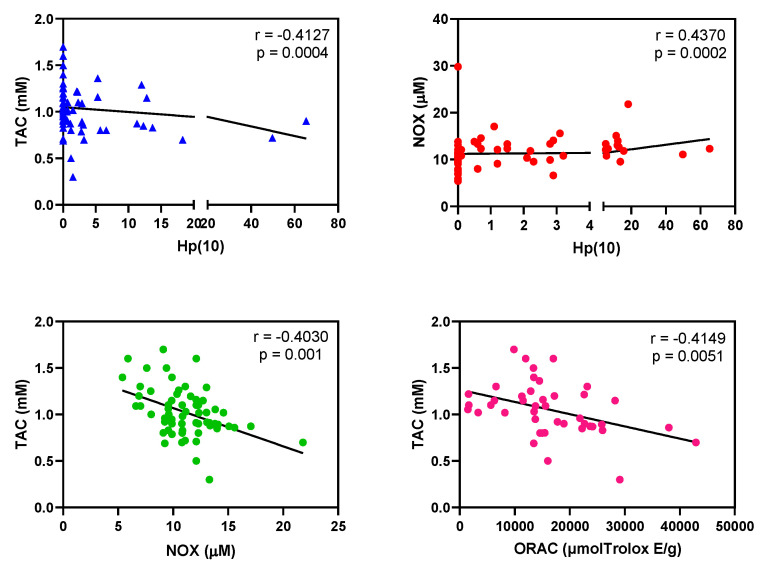
Correlations between cumulative personal dose equivalent Hp(10) and plasma antioxidant-oxidant markers from healthcare workers occupationally exposed to low doses of ionizing radiation. Total antioxidant capacity (TAC), and nitrites (NOX) content were determined from plasma, dietary antioxidant capacity (ORAC) were obtained after analyzing 24-h recall questionnaires and cumulative (Hp(10) was collected. Spearman Spearman’s rank correlation coefficient was used to detect association between dose and redox parameters or between redox parameters. r: Spearman coefficient; p: p-value. For cumulative Hp(10) values = 0 correspond to those participants whose personal physical dosimeter does not reach the minimum recording levels of 0.1 mSv.

Firstly, we analyzed raw data to discern whether values of each variable (TAC, EC-SOD, NOX, TBARS and GSH/GSSG ratio) were different between non-exposed and all the healthcare workers exposed to LDIR. As shown in Figure 1, workers exposed to LDIR had lower TAC values and higher NOX values than non-exposed workers (in both cases, *p* < 0.0001 and *p* < 0.001, respectively, Mann–Whitney test). Besides, the Spearman rank correlation test suggested correlations between the doses, TAC and NOX values (Figure 2). TAC negatively correlated with cumulative Hp(10), NOX positively correlated with cumulative Hp(10) and NOX and TAC showed a negative association. In addition, we found that high ORAC values negatively correlated with TAC. Due to the unexpected negative correlation between TAC and ORAC values for all healthcare workers, we analyzed this association among non-exposed and among exposed workers. We found that the negative correlation occurred only among exposed workers (*p* = 0.09), but not among non-exposed (*p* = 0.68).

### 3.3. Prominent Imbalance of Redox Status in Workers from Nuclear Medicine

To evaluate all the possible factors that could influence redox status (gender, smoking habit, age cholesterol and ORAC scores) in non-exposed and exposed workers to LDIR, we performed a robust statistical analysis considering them (ordinal regression models, Table 2). Analysis revealed no statistical evidence for age effect among groups. However, there was a significant gender effect for NOX (*p* = 0.035) and GSH/GSSG ratio (*p* = 0.034). Females showed lower values of these markers than males. When the three groups of exposed workers were considered separately, TAC values were lower in all exposed groups than in non-exposed workers (*p* = 0.025, *p* = 0.017, *p* = 0.002 for Group 1, 2, and 3, respectively) (Figure 1 and Table 2). Smoking habit and elevated ORAC values significantly reduced TAC values (*p* < 0.001 and *p* = 0.006, respectively). Ec-SOD activity tended to decrease in Group 3 and 1 (*p* = 0.1 and *p* = 0.16, respectively) compared to the non-exposed group (Figure 1). GSH/GSSG ratio was significantly higher in Groups 1 and 2 (*p* = 0.023, and *p* = 0.031). This increase was almost significant for Group 3 (*p* = 0.084) (Figure 1).

A significant increase of TBARS was observed in Group 3 (*p* = 0.008) compared to non-exposed Group. However, TBARS significantly decreased in Group 1 (*p* = 0.015) compared to non-exposed Group. NOX significantly increased in Group 2 (*p* = 0.002) and to a lesser extent, and almost significant, in Group 3 (*p* = 0.054) compared to non-exposed Group (Figure 1).

**Figure 3:**
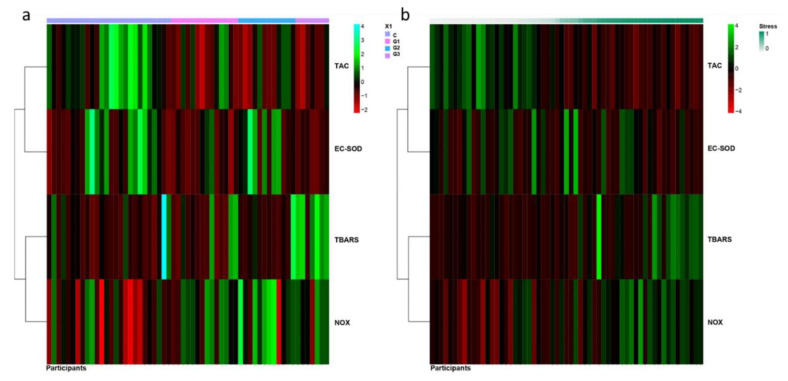
Heatmap representations showing a fuzzy clustering algorithm for blood antioxidant-oxidant markers in hospital workers occupationally exposed to low doses of ionizing radiation and non-exposed hospital workers. Each row represents a redox variable (TAC, EC-SOD, TBARS, NOX) and each column represents a sample from a healthcare worker clustered by the group they belonged (C, G1, G2, G3) (**a**) or by the probability to belong to group 0 (low oxidative stress) or to group 1 (high oxidative stress) (**b**). The dark green to dark red colors corresponds to high to low values. In general, non-exposed workers (C) showed the lowest values of oxidative stress markers and the highest values of antioxidant markers. Workers from Nuclear Medicine (G3) showed the highest values of oxidative stress markers and the lowest values of antioxidant markers (**a**). Workers classified as with higher oxidant status show low TAC) and EC-SOD and high TBARS and NOX levels (**b**). Individuals classified as with lower analysis oxidant status show low NOX and TBARS levels and high TAC and EC-SOD. Beta regression was used to assess differences in the fuzzy clustering results among the different group. TAC: total antioxidant capacity, EC-SOD: extracellular superoxide dismutase activity, TBARS: thiobarbituric acid reactive substances, NOX: nitrites and nitrates. C: non-exposed workers, G1: Interventional Radiology and Cardiology group, G2: Radiation Oncology group, G3: Nuclear Medicine group.

Besides, to perform a more comprehensive and integrative study of the redox status, the probability of the workers to belong to a group with high (1, high TBARS/NOX and low TAC/EC-SOD values) or low (0, low TBARS/NOX and high TAC/EC-SOD values) oxidative stress was modeled as described in the Method section (Figure 3). GSH/GSSG ratio was not included in this analysis because of the number of missing values in several participants. In Figure 3a, the heatmap represented the values of redox markers for each participant clustered by the group they belonged. However, in Figure 3b the heatmap represented the values of redox markers for each participant clustered by the probability to belong to group 0 (low oxidative stress) or to group 1 (high oxidative stress). The results revealed that Groups 2 and 3 had a high probability to belong to the group of high oxidative stress (*p* = 0.048, *p* = 0.01, respectively) (Figure 3b). For Group 1, the probability to belong to high oxidative stress was slightly but not statistically significant (*p* = 0.133) (Figure 3b). Moreover, smoking habit was a factor for high oxidative stress status (*p* = 0.024). Besides, this analysis suggested that females had less probability of belonging to the group of high oxidative stress (*p* = 0.033).

## 4. Discussion

Ionizing radiation has direct and indirect effects on living cells. Direct effects are consequence of the direct interaction of radiation with the atoms of biological molecules such as DNA, proteins or lipids. Indirect effects are consequence of the interaction of radiation, usually with water molecules, leading to free radical formation. The biological effects of ionizing radiation depend, among other factors, on received dose, absorbed dose and the exposure time. While the biological impact of exposure to high dose ionizing radiation has been well documented, the consequences of low dose exposure are still under a considerable debate [24].

Ionizing radiation modulates the redox status via free oxygen radical generation [12,20,25] which, in turn, promotes oxidative stress. The effect of exposure to LDIR in workers on antioxidant response including TAC, GSH, thiol groups and SOD or catalase (CAT) activities has been previously studied [1,7,25,26,27]. However, results remained controversial. Some authors have reported a significant increase of TAC or SOD activity in ionizing radiation-exposed workers compared to non-exposed in different samples such plasma or erythrocytes [1,7,25,26,27]. However, Klucinski et al. (2008) found reduced SOD and CAT activities in erythrocytes of workers operating X-ray equipment [20]. Olisekodiaka et al. (2009) did not find significant differences in antioxidant response in exposed workers compared to their corresponding non-exposed [11]. Malekirad et al. observed higher TAC levels in members from the Radiology Unit than in non-exposed workers [7]. On the other hand, Hagelstrom et al. (1995) found a significantly reduced TAC in workers exposed to LDIR, probably due to the common lack of radiation protection measures applied at that time [28].

In this study, we try to shed some light on the effect of low dose exposure to ionizing radiation on plasma redox status of healthcare workers. We assessed whether healthcare workers exposed to LDIR present differences in blood redox status, which could be consequence of adaptive responses. Besides, we analyzed whether differences in the redox status could be influenced by personal dose exposure, or by their dietary antioxidant capacity.

We observed that workers from Nuclear Medicine (Group 3) presented the highest values of Hp(10)_12_ and ORAC values (Table 1). Our results corroborated some previous studies suggesting a redox imbalance in the exposed population to LDIR [1,11,12,29]. To improve the current study, we also analyzed the effect of dose and antioxidant dietary intakes in this imbalance. Besides, we included confounding factors that could misunderstand the findings like gender, age, tobacco, or cholesterol. We found an putative association between high cumulative Hp(10) and low plasma TAC values in workers occupationally exposed to LDIR, which could suggest a negative long-term effect of the dose on the antioxidant response (Figure 2). However, we should be cautious in drawing conclusions, because we noticed that there were participants with zero as a value of cumulative Hp(10) (Table 2). Besides, several participants presented values of zero for Hp(10)_12_ and Hp(0.07)_12._ We should take into account that the minimum measurable dose or limit of detection for personal physical dosimeters was 0.1 mSv. All workers occupationally exposed to LDIR have health risks but they also have protective measures to reduce the risk. In general, Group 1 receive higher doses than Group 2 and 3. However, Group 1 have more protective measures, formative programs, etc. than the other Groups. Group 2 use much automatized techniques thus receiving lower doses than Group 1 and 3. Finally, Group 3 is the least radio-protected group and has a double risk: irradiation and contamination. Therefore, it is necessary to increase the number of participants with high personal dose equivalent to confirm this association.

**Figure 4:**
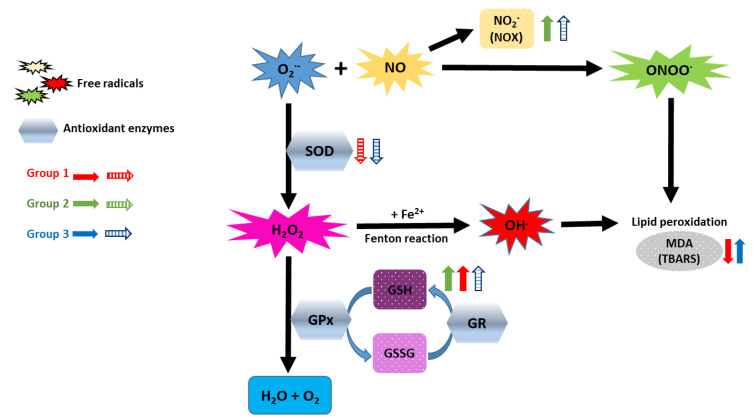
Summary of plasma antioxidant-oxidant status in healthcare workers occupationally exposed to low doses of ionizing radiation (LDIR). Oxidative stress arises from an imbalanced redox status between the production of free radicals such as O_2_^•−^, ^•^OH, H_2_O_2_, NO, ONOO^•^, and the antioxidant defense machinery such as SOD, GPx or GSH to remove them. Free radicals can randomly react with lipids leading to lipid peroxidation and MDA formation (TBARS), proteins leading to, among others, protein nitration or nucleic acids. Hospital workers exposed to LDIR (G1: Interventional Radiology and Cardiology, G2: Radiation Oncology, G3: Nuclear Medicine) present a redox imbalance in plasma. Lower antioxidant response (TAC and SOD activity) and higher oxidative stress (NOX and TBARS) was found, especially for G3. Low SOD activity could be responsible for accumulation of O_2_^•−^, NOX and even lipid peroxidation. On the other hand, high GSH could be an adaptive response to oxidative stress that could help to remove free radicals (e.g., H_2_O_2_). Total antioxidant capacity (TAC) is a measurement reflecting the antioxidant response or status of a biological sample. All three groups showed lower TAC than controls. Solid arrows represent statistical decreases/increases and dashed arrows tendencies to decrease/increase. O_2_^•−^ superoxide; ^•^OH: hydroxyl radical; NO: nitric oxide; NOX: nitrites; ONOO^•^: peroxynitrite; GSH: glutathione; GPx: glutathione peroxidase; GR: glutathione reductase.

When we focused on different services, we detected that the effect was a bit different depending on the hospital service to which they belonged. In general, blood antioxidant response (TAC and EC-SOD) seemed to be reduced in healthcare workers exposed to LDIR, especially for Group 3 and lesser extent to Group 1 and 2 (Figure 1). Oxidative stress markers (TBARS and NOX) increased especially for Group 3 and lesser extent to Group 2. In this case, a reduction in TBARS values was found in Group 1 (Figure 1). A schematic summary of these results is shown in Figure 4. In general antioxidant response was clearly reduced on Group 3, the one presenting the higher levels of radiation-exposure. The differences between Group 1 and 2 could suggest that discrepancies among different studies could be attributed to the different occupationally exposed groups analyzed. The beta regression model confirmed these results obtained with ordinal regression models (Figure 3). This analysis indicated that the probability of belonging to a group with oxidative stress (imbalance between antioxidant response and free radicals formation) was high for Nuclear Medicine Service (Group 3), medium for Radiation Oncology Service (Group 2) and low for Interventional Radiology and Interventional Cardiology Service (Group 1) (Figure 3). In particular, we observed reduced TAC levels for all analyzed groups exposed to ionizing radiation. EC-SOD activity tended to decrease in workers from Interventional Radiology and Cardiology Service (group 1) and from Nuclear Medicine Service (group 3) (Figure 1).

Our opposite findings in TAC values respect to other studies could be due to differences on the studied cohort, the equivalent dose measurements, the type of samples, or even to the methods to measure “antioxidant capacity”. TAC values could decrease due to aging, radiation exposure, smoking habit, antioxidant consumption, etc. [30,31,32]. Therefore, we took into account these variables in our study. In particular, smoking habit significantly reduced TAC values and increased the probability to present a high oxidative stress status in plasma (Table 2). Tobacco contains many harmful compounds, such as nicotine, nitrous oxide, cadmium, ethanol, etc., that may react with biological macromolecules and increase free radicals formation. Many reports show that smoking induces oxidative stress in humans [33,34,35].

We also noticed gender differences on redox status supporting those found by other authors [36]. Females were less susceptible to oxidative stress than males (Table 2). Among others, estrogen levels may contribute to these gender differences because of their antioxidant properties [37]. Our analysis showed that females had lower NOX values and lower probability to present a high oxidative status than males for all groups. So, we considered gender, age, dietary antioxidant capacity (ORAC value), smoking habit and Hp(10) as confounding factors in our statistical analysis.

Healthcare workers from Interventional Radiology and Interventional Cardiology Service (Group 1) and from Radiation Oncology Service (Group 2) presented significant higher GSH/GSSG ratio than non-exposed (Figure 1). These results are similar to other findings in healthcare workers occupationally exposed to ionizing radiation [27,38,39]. Elevated GSH levels in blood could be an adaptive response to oxidative stress in order to protect cells from genetic alterations. However, as above-mentioned, we had several missing values to a obtain robust conclusion with this variable. On the other hand, other authors did not find changes of GSH in blood of workers exposed to low levels of ionizing radiation [26].

Concerning oxidative stress markers (NOX and TBARS), we found a positive relationship between high cumulative Hp(10) and high NOX in blood of workers occupationally exposed to LDIR (Figure 2). NOX significantly increased in workers from Radiation Oncology Service (Group 2). NOX are stable metabolites of NO, and consequently their presence might be due to high levels of NO in the sample. NO can react with the free radical superoxide to form peroxynitrite, a potent oxidant that can damage a wide variety of cellular molecules including DNA or proteins (e.g., formation of nitrotyrosine) (Figure 4). High content of intracellular NO was detected in peripheral blood lymphocytes from workers of atomic industry [40]. Our data from TBARS (marker of MDA formation) content partly corroborated those found by others authors respect to high MDA levels [7,26]. We detected elevated levels of TBARS in healthcare workers from Nuclear Medicine Service (Group 3), but not in workers from the Radiation Oncology Service (Group 2). Conversely, workers from the Interventional Radiology and Interventional Cardiology Service (Group 1) showed significant decrease in TBARS (Figure 1). More studies are needed to clarify this decrease on lipid peroxidation. Nuclear Medicine workers with a decreased antioxidant response could have a higher concentration of oxidant species in the medium responsible for cell membrane lipids oxidation (high values of TBARS).

In our study, we detected that dietary antioxidant capacity (ORAC) was significantly higher in workers from Nuclear Medicine Service (Group 3) than in the non-exposed workers (Table 1). Besides, this group presented the lowest values of TAC and the highest values of ORAC. Surprisingly, we found a significant correlation between high ORAC scores and low TAC values (Figure 2). In spite of having the highest values of ORAC in their diet, healthcare workers from Nuclear Medicine Service showed the highest level of oxidative stress. Currently there is still limited information available on whether different antioxidant status in body fluids properly reflects a dietary intake of antioxidants [41,42]. Some authors have found that antioxidant dietary intake does not affect plasma TAC in old population [43]. Perhaps we had expected that dietary intakes (ORAC values) predicted plasma antioxidant status, as previously described for healthy young population [44,45], or sarcopenic frail elderly patient [46]; however, we found an inverse relationship between plasma TAC and antioxidant dietary intake among exposed workers. We believe that extensive studies are needed to conclude how dietary intakes affects redox status in this population exposed to LDIR. These results could be a consequence, at least partly, of their double risk (contamination and irradiation) and a higher level of occupational exposure of this service compared to other services. It could be that Nuclear Medicine workers are aware of the risks of irradiation and they practice a healthy diet. Perhaps the imbalance redox status would become even higher without a healthy diet in these workers. In general, the present study suggests that healthcare workers occupationally exposed to low ionizing radiation could be more concerned about adopting healthier nutrition than non-exposed workers. Healthcare workers continuously exposed to LDIR could increase their oxidative stress levels [47]. We hypothesize that an antioxidant, anti-inflammatory and immune stimulant-enriched diet could counteract oxidative stress. For instance, fruits and vegetables are “antioxidant” foods as “gold standards”, so it is recommended to eat at least five servings a day. Within fruits, the most antioxidant of these are those present in the Mediterranean Diet and tropical diet [48]. Vegetables such as green leaves, cruciferous, tomato, onion and garlic [49], and Mediterranean spices [50], dark chocolate [51], green tea [52] or turmeric [53] are some examples of important sources of antioxidants.

## 5. Conclusions

In summary, we present a study based on an exhaustive statistical analysis including several confounding factors that allowed us to get robust conclusions. Our findings suggest that long-term exposure to LDIR may induce imbalanced antioxidant response decreasing it and an increase in oxidative stress markers (O_2_^•−^, MDA). We hypothesized that the degree of downregulation of antioxidant response should depend on the dose they received for belonging to a particular service. The obtained data that workers from Nuclear Medicine Service had the worst redox status compared to healthcare workers at the Interventional Radiology and Interventional Cardiology and Radiation Oncology Services. Moreover, it is important to consider other factors, such as tobacco, diet, partial and non-homogeneous body exposure and contamination risk.

We are fully aware that future studies will help to clarify the effect of LDIR exposures increasing number of participants, oxidative stress markers, etc. Besides, they will help to prevent or/and treat oxidative stress (changes in diet or radiation protection measures) and possible health complications.

## Figures and Tables

**Table 1:** Characteristics of participants included in the study.

Characteristics	Control	Group 1	Group 2	Group 3
**N of subjects**	28	14 9 nurses, 4 physicians, 1 radiographer	18 6 nurses, 6 physicians,3 physicists, 3 radiographers	10 5 nurses 1 physician 1 pharmacist 3 radiographers
**Males**	10	3	8	1
**Females**	18	11	10	9
**Age (yr)** Mean (SEM)	43 (2)	48 (1)	50 (3)	48 (1)
**Smokers** (n smokers/group)	3/28	4/14	4/18	5/10
**High Cholesterol** (n subjects with >220 mg/dL/group)	6/28	1/14	0/18	1/10
**Hypertension** (n of subject with ≥140/90 mm Hg/group)	1/28	2/14	0/18	0/10
**Vitamins/minerals supplements** (n of subject/group)	1/28	1/14	0/18	0/10
**ORAC (μmol TE/100 g)** Mean (SEM)	12599 (1449)	17796 (2615)	17272 (3039)	23368 (3716) *
		19074 (1822) (Groups 1–3)		
**Hp(10)_12_ (mSv)** Mean (SEM) [Range]	----	0.22 (0.15) [0.0–1.9]	0.05 (0.03) [0.0–0.6]	0.45 (0.17) ^a,b^ [0.0–1.4]
		0.2 (0.07) [0.0–1.9] (Groups 1–3)		
**Hp(0.07)_12_ (mSv)** Mean (SEM) [Range]	----	11.05 (5.66) [1.8–38.7]	0.12 (0.08) ^c^ [0.0–0.5]	3.14 (1.46) [0.0–11.1]
		4.7 (2.0) [0.0–38.7] (Groups 1–3)		
**Cumulative Hp(10) (mSv)** Mean (SEM) [Range]	----	5.16 (1.43) [0.0–13.7]	5.56 (2.67) [0.0–49.8]	9.8 (6.4) [0.0–65.3]
		6.44 (1.95) [0.1–65.3] (Groups 1–3)		

**Note:** Hp(10)_12_: Personal dose equivalent during 12 months; Hp(0.07)_12_: Hand skin dose equivalent during 12 months; SEM: standard error of the mean; TE: Trolox equivalent; ORAC: Oxygen Radical Absorbance Capacity. * *p* < 0.05 group 3 vs. control; ^a^ *p* < 0.05 group 3 vs. group 1: ^b^ *p* < 0.01 group 3 vs. group 2; ^c^ *p* < 0.05 group 2 vs. group 1. ANOVA-one way and post hoc Tukey test for ORAC and Kruskal–Wallis and Dunn’s multiple comparisons test for Hp(10)_12_ and Hp(0.07)_12_. Control: Non-exposed workers; Group 1: Interventional Radiology and Cardiology; Group 2: Radiation Oncology; Group 3: Nucleoracar Medicine.

**Table 2:** Estimates, standard errors and p-values of the ordinal regression models for each redox parameter.

Variables	Estimate	S. Error	*p*	Estimate	S. Error	*p*	Estimate	S. Error	*p*	Estimate	S. Error	*p*	Estimate	S. Error	*p*
	TAC			EC-SOD			GSG/GSSG			TBARS			NOX		
Age	0.043	0.028	0.126	0.026	0.029	0.371	−0.032	0.041	0.426	−0.009	0.029	0.763	−0.004	0.029	0.888
Gender	0.749	0.626	0.232	−0.202	0.672	0.764	−2.745	1.293	0.034	−0.302	0.707	0.669	−1.385	0.658	0.035
Tobacco	−3.455	0.929	<0.001	0.51	0.837	0.542	−0.637	1.673	0.703	0.874	0.898	0.33	0.905	0.781	0.247
High Cholesterol	1.455	0.979	0.137	−0.043	0.956	0.964	2.138	1.27	0.092	−0.978	0.927	0.291	1.136	0.932	0.222
ORAC	−0.102	0.037	0.006	0.022	0.035	0.525	0.016	0.079	0.844	0.011	0.038	0.772	0.028	0.036	0.443
C vs. Group 1	−2.364	1.058	0.025	−1.276	0.919	0.165	4.983	2.186	0.023	−2.34	0.959	0.015	1.276	0.927	0.169
C vs. Group 2	−2.009	0.841	0.017	0.87	0.818	0.288	4.031	1.868	0.031	0.081	0.743	0.913	2.701	0.893	0.002
C vs. Group 3	−3.176	1.032	0.002	−1.696	1.031	0.1	2.769	1.604	0.084	4.428	1.682	0.008	1.845	0.959	0.054

C: Non-exposed workers; Group 1: Interventional Radiology and Cardiology; Group 2: Radiation Oncology; Group 3: Nuclear Medicine; TAC: total antioxidant capacity; EC-SOD: extracellular superoxide dismutase activity; GSH/GSSG: glutathione/oxidized glutathione ratio TBARS: thiobarbituric acid reactive substance, NOX: nitrites and nitrates.

## Supplementary Materials

The following are available online at https://www.mdpi.com/2076-3921/9/9/778/s1, Table S1: Personal dose equivalent of healthcare workers occupationally exposed to LDIR.
