# Supplementary material for: Redox Status, Dose and Antioxidant Intake in Healthcare Workers Occupationally Exposed to Ionizing Radiation
Source: Antioxidants (Basel). 2020 Aug 21;9(9):778. doi: 10.3390/antiox9090778 (PMC7554777; doi:10.3390/antiox9090778)
Supplement: Supplementary file 1 [file antioxidants-09-00778-s001.pdf]

Table 1. Personal dose equivalent of healthcare workers occupationally exposed to LDIR.

	$Hp(10)_{12}$ (mSv)	$Hp(0.07)_{12}$ (mSv)	Cumulative Hp(10) (mSv)	Cumulative exposure duration (months)
**Group 1 14 participants**	0.0	---	0.0	39
	0.0	---	13.7	373
	0.0	3.8	12.8	325
	0.0	--	0.1	53
	1.2	6.9	5.3	176
	1.9	38.7	5.7	82
	0.0	1.8	0.0	9
	0.0	---	12.3	445
	0.0	---	2.8	186
	0.0	4.7	2.3	446
	0.0	10.4	5.3	157
	0.0	---	12	442
	0.0	---	0.0	81
	0.0	---	0.0	11
**Group 2 18 participants**	0.0	---	1.1	189
	0.6	0.5	49.8	369
	0.0	0.0	1.2	140
	0.0	---	0.7	54
	0.0	---	0.0	81
	0.0	---	2.2	180
	0.0	---	2.9	275
	0.0	0.0	11.3	397
	0.0	0.0	3.1	235
	0.0	---	2.9	277
	0.0	0.0	2.1	106
	0.0	0.2	15.7	336
	0.0	---	0.0	19
	0.0	---	1.5	194
	0.24	---	2.3	209
	0.0	---	2.8	282
	0.0	---	0.6	103
	0.0	---	0.0	82
**Group 3 10 participants**	0.0	---	6.6	275
	0.1	---	3.2	148
	1.4	4.8	0.1	67
	0.2	---	0.6	166
	0.7	2.7	0.5	105
	0.7	2	65.3	447
	0.1	0.1	0.7	139
	1.3	11.1	18.3	170

	0.0	1.3	1.5	166
	0.0	0.0	1.2	38

Note: Healthcare workers occupationally exposed to LDIR whose personal dosimeter dif not reach the minimum recording levels of 0.1 mSv presented a value of 0.0. (---): No data obtained
